# Family functioning following a brief, virtual emotion‐focused family therapy intervention for children's mental health

**DOI:** 10.1002/jcv2.70074

**Published:** 2026-01-13

**Authors:** Laura Colucci, Mirisse Foroughe, Imogen M. Sloss, Dillon T. Browne

**Affiliations:** ^1^ University of Waterloo Waterloo Ontario Canada; ^2^ Family Psychology Centre Toronto Ontario Canada

**Keywords:** child mental health, EFFT, emotion‐focused, family functioning, family therapy, parenting

## Abstract

**Background:**

Brief emotion‐focused family therapy (EFFT) interventions have demonstrated numerous positive outcomes across the domains of child mental health and parent psychosocial well‐being. However, there is limited research examining interpersonal processes at the family‐level of analysis following 2‐day EFFT programs.

**Methods:**

This study explored family functioning in the year following a virtual, parent‐focused EFFT intervention (*n* = 159 caregivers, representing 124 families and 264 children). Caregivers completed the General Functioning subscale of the Family Assessment Device at 6 timepoints from baseline to 12‐month post‐intervention. Multilevel modeling was used to complete growth curve analysis, exploring post‐intervention changes in family functioning over time. This allowed for the exploration of between versus within family differences pre‐ to post‐intervention in the study sample.

**Results:**

Variance in family functioning was attributable to stable differences between caregivers (level 2; 59%) and change over time (level 1; 41%), including measurement error. Growth curve analysis identified positive changes in family functioning post‐intervention, with a cubic trajectory of improvement. Higher COVID‐19 disruption, caregiver psychological distress, and parenting stress significantly predicted lower baseline family functioning, but did not significantly interact with change over time.

**Conclusions:**

These results suggest that there is a general pattern of non‐linear change following EFFT workshops, specifically relating to family interpersonal dynamics. Overall, the study findings expand the evidence base for this promising, brief, relational intervention, now available in virtual formats, thereby increasing access for busy families.

## Key Points

**What's Known?**

- Brief and virtual emotion‐focused family therapy workshops are an accessible, relationally‐focused parenting intervention for children's mental health recovery.

**What's New?**

- This study presents the first evidence that this program is associated with change at the family level in the area of family functioning and the interpersonal climate of the home; which is linked to pandemic disruption, family size, parent ps distress, and parenting stress.

**What's Relevant?**

- Results affirm the necessity of a multi‐level family systems framework when evaluating outcomes from parent‐involved and emotion‐focused children's mental health care interventions.

## Graphical abstract

This study presents the first evidence that brief emotion‐focused family therapy (EFFT) is associated with change at the family level in the area of family functioning; a construct that is also associated with pandemic disruption, family size, parent psychological distress, and parenting stress. Results affirm the necessity of a multi‐level family framework when evaluating outcomes from children's mental health programs.

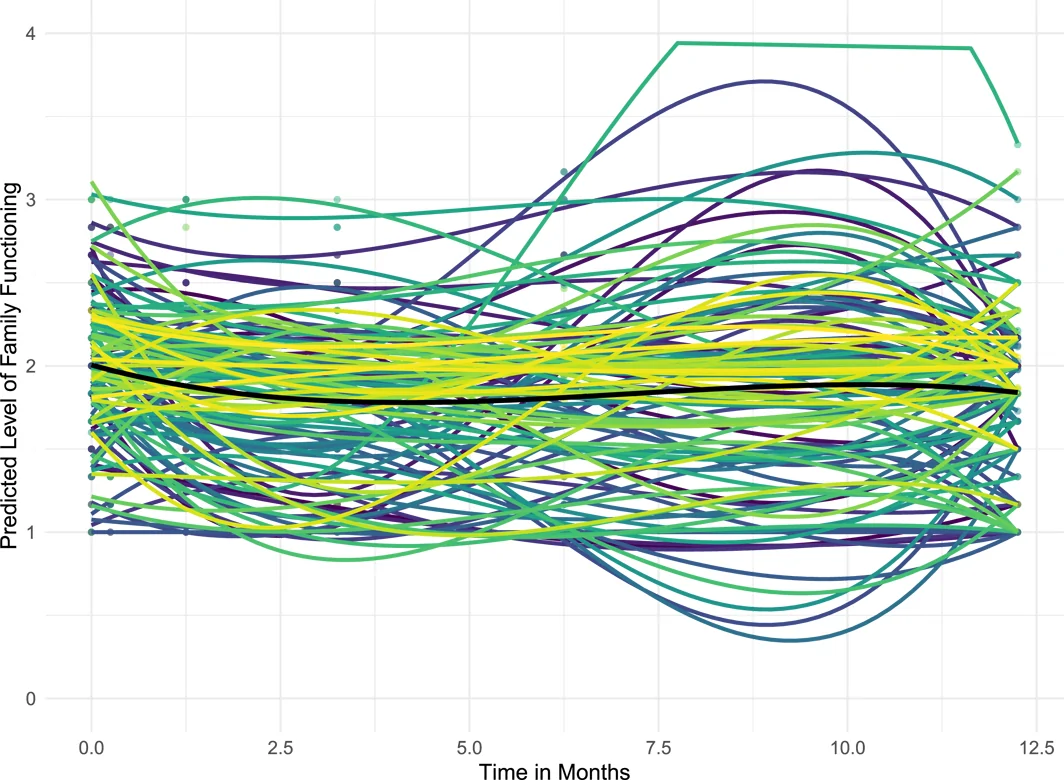

## INTRODUCTION

Family systems theory emphasizes the interconnectedness of individuals and relational subsystems within the family unit (e.g., between parents, the parent‐child relationship, and sibling dynamics; Browne et al., 2015; Cox & Paley, 1997, 2003). In this way, children and caregivers are connected in their experiences of psychological distress, as their affective states emerge and ‘spillover’ across these relational levels (Nelson et al., 2009; Staccini et al., 2014). Thus, when exploring intervention outcomes from children's mental health services (especially those with a parent‐focused component), family‐wide factors should be an included outcome. Emotion‐focused family therapy (EFFT) is an emerging and promising therapeutic approach for children's mental health care where parent involvement is central (LaFrance et al., 2020). Brief, group EFFT workshops for caregivers offer an efficient and accessible parent‐focused intervention for transdiagnostic child mental health challenges. Existing research has demonstrated positive results following this intervention across child and parent outcomes, including children's behaviour problems and caregiver self‐efficacy (Cordeiro et al., 2022; Foroughe et al., 2019, 2023). However, no studies have explored family functioning as an outcome following brief EFFT workshops, and caregiver psychological distress has also been largely overlooked in the study of this approach; the paucity of research on these constructs in relation to EFFT represents a limitation in the current evidence base for this intervention, as EFFT techniques are encouraged to generalize across family members (LaFrance et al., 2020). This study sought to address these gaps through a longitudinal analysis of family functioning in the year following a virtual, two‐day, group, parent EFFT program.

### Caregiver psychological distress in the context of children's mental health

Parent mental health and psychological distress are among the most studied predictors of children's psychological well‐being. Research has demonstrated robust linkages between these constructs and children's mental health (both negative affect and behaviour problems), the parent‐child relationship, and children's own trajectories of psychological distress (Goodman et al., 2011; Kamis, 2021; Smith, 2004). Parent psychological distress also plays a significant role in informing the effectiveness of parenting interventions for children's mental health (Ansar et al., 2023; Maliken & Katz, 2013). Moreover, it is also related to the quality of parenting practices, for better and for worse, within the parent‐child relationship and can inform the degree of family‐wide stress during crises (Borden et al., 2014; Masarik & Conger, 2017). Non‐clinical (naturalistic) studies have demonstrated that parent psychological distress is a predictor of family functioning, as well (Pu & Rodriguez, 2024). Relatedly, caregiver psychological distress was negatively impacted during the initial years following the COVID‐19 pandemic, with studies identifying higher rates of parenting stress and mental health concerns, which predicted deteriorations in parenting behaviours (Berg‐Nielsen et al., 2002; Duchovic et al., 2009; Thomson et al., 2023; Yakub et al., 2024). Altogether, these findings are consistent with the family stress model, suggesting that caregiver distress is a multisystemic family phenomenon, arising from complex interactions between environmental, individual‐specific, and family‐wide factors, ultimately casting a deleterious influence on children's developmental outcomes, broadly defined (Masarik & Conger, 2017). Thus, caregiver psychological well‐being is an important factor to consider in the context of family‐centered mental health interventions for children.

### Expanding outcomes in EFFT research

In recent years, there has been a proliferation of emotion‐focused group parent training programs including brief EFFT, Parent‐Child Interaction Training, and Tuning in to Kids, among others (Havighurst et al., 2020). These approaches have been developed in response to calls for children's mental health care practitioners to apply family‐wide, transdiagnostic, and trauma‐informed lenses in their work, so to maximize family resources in the recovery process (Buka et al., 2022; Everett et al., 2021). Following COVID‐19, this arm of children's mental health services also experienced the rapid development of virtual interventions, in order to maximize service accessibility during a period where there was a population‐wide increase in the incidence of children's mental health disorders (Foroughe et al., 2021; Spencer et al., 2020).

EFFT is a relationally‐focused and parent‐involved intervention for children's mental health problems that emphasizes the role of parent‐child co‐regulation (LaFrance et al., 2020). This transdiagnostic approach can be used for a wide variety of presenting concerns across childhood, adolescence, and young adulthood. Designed to be used as a standalone treatment or in tandem with other services, brief, two‐day parent EFFT workshops are one highly accessible and increasingly common format of this intervention (Foroughe et al., 2019). Research to date on EFFT has shown dynamic and enduring treatment gains, which remain up to a year following the program across children's mental health, caregiver self‐efficacy, caregiving blocks, and positive parent perceptions of child negative affect (Cordeiro et al., 2022; Foroughe et al., 2019, 2023; Goveas et al., 2024; LaFrance et al., 2020; Wilhelmsen‐Langeland et al., 2019).

Based on a review of emotion‐focused parenting interventions, parent mental health is often included as distal intervention outcome in child mental health programs of this nature; however, the broader family interpersonal context is seldom considered, and more research is needed to clarify how parent well‐being and family‐level outcomes may jointly fluctuate with one another (Havighurst et al., 2020). These limitations have recently gained empirical attention within EFFT research. For example, in a case study of longer‐term virtual EFFT with co‐parents of a child with behavioural difficulties, positive change in family functioning was observed over time (Smith et al., 2023). Further, improvements have also been observed for caregiver resilience following EFFT workshops (Sloss et al., 2025). These results are promising, and they suggest that there may be unidentified family‐wide benefits associated with brief and virtual EFFT programs, representing another possible intervention target.

### Family functioning following caregiver‐focused interventions

Family relationships are inherently bidirectional interactional systems. Research has established that the ‘whole family’ (or the interpersonal environment created by all family members) represents its own unique level of analysis; thus, family functioning represents an outcome that may be indirectly targeted by parenting interventions (Cox & Paley, 2003; Eichelsheim et al., 2009). Multi‐level family research on the parent‐child relationship has identified that, despite the bidirectional effects that caregivers and children have on one another, parents may have greater influence over the interpersonal tone of the family home than do children (Browne et al., 2015; Sokolovic et al., 2020). In this way, just as negative parenting practices, caregiver distress, and child mental health challenges can create threats to family‐wide well‐being, so might emotion‐focused parenting interventions represent an opportunity to mitigate caregiver distress, support positive parenting, and strengthen family relationships. In essence, by teaching parents advanced caregiving techniques in response to children's mental health concerns, these programs may offer benefits that extend across multiple relational layers within the family system (Havighurst et al., 2020; Nelson et al., 2009; Zahl‐Olsen et al., 2023).

### The current study

To address these gaps in the literature, this project explored changes in family functioning in the year following a brief and virtual parent‐focused EFFT intervention. After a review of existing research, several hypotheses were developed: (H1) there will be stable between‐family and within‐family differences in family functioning, (H2) on average, families will improve over time, though there will be substantial between‐family differences in the rates and patterns of improvement, (H3) lower COVID‐19 disruption, female gender, dual‐caregiver participating families, and fewer children in the home will predict higher baseline family functioning, and (H4) higher caregiver psychological distress and parenting stress will predict lower baseline family functioning, and more rapid improvement over time (Colucci et al., 2023; Foroughe et al., 2019; Georgiades et al., 2008; Jenkins et al., 2009; Sloss et al., 2025; Smith et al., 2023).

## MATERIALS AND METHODS

### Participants

The study sample includes a longitudinal cohort of caregivers from a major city in Ontario, Canada (*n* = 159 caregivers, representing 264 children across 124 families) who participated in virtual two‐day EFFT workshops between September 2020 and November 2021. All program participants were invited to the study; accordingly, this sample includes 35 caregivers who were nested within the same family and reported on the same children. A small number of participants (6%) withdrew from the study for reasons including: dissatisfaction with the questionnaires and/or intervention, having no contact with their child, and/or change of interest in participating. Less than 10% had atypical participation (e.g., partial workshop completion; for more details, see Appendix S1). Participating caregivers were asked to report child age, gender, presenting concerns, and history of intervention for all children in the home (up to four per family), regardless of their need for mental health services.

The average age of the child for whom parents were primarily seeking services (the target child) varied across the sample (*M =* 12.44 years, SD = 4.80; range: 1.28–29.90). The average participating caregiver had two children (*M* = 2.11, SD = 0.77). The subsequent demographics reflect data from a single caregiver per family, where the target child was aged 5–25 years (*n* = 117; see Appendix S2). While this range remains large, it also reflects a significant strength of the EFFT model, as skills translate across the lifespan (not only to children of different ages, but also to spouses, parents, in‐laws, and so on).

Target children included boys (48%), girls (46%), and transgender (<1%) and gender non‐conforming children (5%). Parents identified concerns for their child (all that apply), including: anxiety (76%), depression (46%), eating disorders (15%), behavioural dysregulation (55%), emotional/social/relationship concerns (68%), autism (6%), ADHD (32%), cognitive difficulties (11%), somatic complaints (15%), stress (43%), self‐esteem challenges (60%), medical conditions (9%), abuse/trauma (14%), or other (9%). Parents reported if their child had received any DSM‐5 diagnoses (42%) or previous mental health treatment (70%, range = 1–300 sessions, *M* = 21, SD = 40); the most common diagnoses were Attention‐Deficit Hyperactivity Disorder, Specific Learning Disorder, Generalized Anxiety Disorder, and (unspecified) depressive disorder.

Caregivers reported their gender; potential responses included woman, man, gender variant/non‐conforming, trans male, trans female, or other (please specify). One participant identified as gender variant/non‐conforming; analyses were conducted with and without this caregiver and the pattern of results was identical. In order to include all caregivers in the analysis, a binary variable was created with ‘female’ as the reference category, with ‘male’ and all other gender identities coded as 1. Parents also reported their ethnic background, highest level of education, immigration or citizenship status, relationship status, home status of and relationship to the represented children, the number of children under 18 in the home, and monthly household income bracket ($0–$1600 to $8300+/month; Median = $6601–$8300 CAD). See Table 1 for other demographic information.

**TABLE 1:** Demographic variables for the study sample.

Caregiver	%	Caregiver	%	Caregiver and child	%
Gender		Citizenship		Relation to children	
Female	89	Canadian citizen	94	Children by birth with current partner	73
Male	11	Immigrant	2		
Gender variant or gender non‐conforming	1	Permanent resident	3	Blended family‐Child related to one parent	22
Ethnicity		Non‐immigrant‐Student or work visa	1	Children by adoption	5
White	74	Other	1	Extended family or kinship	3
Black	3	Monthly income		Target child's home status	
Hispanic	2	$0–$1600	2	Child at home full‐time	82
Pacific Islander	0	$1601–$3300	6	Child at home part‐time	13
West Indian	1	$3301–$5000	21	Child not living at home^a^	5
Middle Eastern	1	$5001–$6600	8	Age of the target child	
East Asian	6	$6601–$8300	22	5–12 years	58
South Asian	11	$8300+	42	13–18 years	38
Indigenous	3	Caregiver relationship status		19+ years	4
Other	6	Single parent	13	No. children under 18 at home	
Education		Opposite‐sex partners‐Living apart	7	One	26
High school diploma or equivalent	4	Same‐sex partners‐Living apart	1	Two	55
Trade certificate/diploma	1	Opposite‐sex partners‐Living together	75	Three	19
College, CEGEP, or college certificate	17	Same‐sex partners‐Living together	1	Four	1
University certificate	4	Other	4		
Bachelor's degree	39				
Certificate or degree above Bachelor's	37				

^a^ Of the four children over 18 years old in the study, two were living at home with the caregiver.

### Procedure

Participants were caregivers seeking services for their children at an outpatient community mental health practice in a large urban centre. Families were contacted through a research flyer or invited to participate following registration for the brief EFFT workshops if they were caregivers of a child of any age, with no other exclusion criteria. This study received clearance through the University of Waterloo Ethics Committee (#41875). Informed consent was obtained electronically. Due to COVID‐19 constraints and the high service needs for children's mental healthcare at the time of the study, all participants received the intervention.

The intervention consisted of a virtual and manualized, two‐day group workshop geared towards developing advanced caregiving skills to support family relationships and children's mental health. The goals of the program were: (1) *emotion coaching:* equip caregivers with the skills to model emotion regulation and assist their child(ren) as emotion coaches, (2) *behaviour coaching:* empower caregivers to become recovery coaches for their children by applying symptom interruption and behavioural intervention skills, (3) *therapeutic apology:* assist parents in strengthening their relational repair skills through a technique called the therapeutic apology, and (4) *caregiver blocks:* provide parents with the opportunity to practice workshop skills and process their own experiences (emotional blocks) that may inhibit the effective implementation of components 1–3. The workshops maintained a consistent format across 11 offerings, were delivered live, and were facilitated by a clinical psychologist who is certified as an EFFT trainer and was directly trained by the cofounders of EFFT. Additional details are discussed in other publications and Appendix S4 (Foroughe et al., 2019; Lafrance Robinson et al., 2014).

### Measures

Caregivers completed questionnaires at pre‐workshop baseline, immediately post‐workshop, and 1, 3, 6, and 12 months post‐workshop (Times 0–5).

#### Pandemic‐related stress

The COVID‐19 Family Stressor Scale (CoFaSS; Prime et al., 2021) is a 16‐item measure assessing pandemic‐related stress, including environmental chaos, income‐related impacts, and family relationships. Items have a three‐point Likert scale from (1) *Not true* to (3) *Very true.* The CoFaSS was measured at Time 0 only and internal consistency was good (*α* = .83).

#### Family relationships

The General Functioning subscale (GF6+) of the McMaster Family Assessment Device (FAD; Boterhoven de Haan et al., 2015) is a six‐item measure that assesses family connectedness and problem solving, on a four‐point scale (*1‐Strongly Disagree* to *4‐Strongly Agree*). Scores were averaged for interpretation (1.0–4.0); scores >2.0 suggest ‘unhealthy’ functioning, and scores <2.0 suggest ‘healthy’ functioning. The internal consistency was good‐excellent across all timepoints (range: *α* = .84–.93).

#### Caregiver mental health

The Kessler Psychological Distress Scale (K10) was used to measure parent psychological stress in the last 30 days, including low mood, nervousness, and somatic symptoms (Kessler et al., 2002). The K10 uses a 10‐item, five‐point Likert scale, from *1‐None of the time* to *5‐All of the time*, with higher scores indicating greater distress (clinical cut‐off: 20). The internal consistency was good‐excellent across all timepoints (range: *α* = .85–.92).

#### Parenting stress

Using a single item, caregivers reported on their level of parenting stress related to the target child in the last two weeks. Responses ranged from *1‐Not at all stressful* to *7‐Extremely Stressful* (Hartley et al., 2016). To maintain the focus on children for whom parents sought services, only data from the target child was used (though the results were the same when an average of parenting stress across all children was tested).

### Data analysis

Multilevel modelling was used to complete growth curve analysis in RStudio with the *lme4* package (Bates et al., 2015). The data had two levels: Repeated measures (Time‐Level 1) were nested within caregivers (Participants‐Level 2). Intra‐class correlation coefficients (ICCs) were used to explore variance partitioning across levels, which were compared across models. Restricted maximum likelihood was used to fit models, which were compared with a likelihood ratio test. An unconditional growth model was used to measure change over time (in months), where the first observation represented pre‐intervention baseline (0 months) and the intervention was completed one week after. Linear models were estimated with a random intercept and fixed effect of time, and then a random intercept and random effect of time. Non‐linear change was explored through quadratic and cubic terms. Subsequently, time‐invariant covariates were added as main effects, followed by time‐varying predictors. Finally, cross‐level interactions were added to explore whether these predictors modified trajectories of change. For more information regarding the use of this approach within family systems research, see Appendix S3.

### Missing data

Univariate outliers (±3 *SD*) were Winsorized (0–2 cases). Using Mahalanobis Distance, one multi‐variate outlier was identified (*p* < .001) and removed, as the pattern of results diverged with its inclusion. Using Little's (1998) Missing Completely at Random (MCAR) test, the MCAR assumption was rejected (*χ* ^2^(240) = 343.90, *p* < .001). This study had very good retention (>86% across the FAD, K10, and parenting stress measure); missing data appeared to be due to attrition, which is common in longitudinal studies of this duration. To adjust for bias due to missingness, non‐parametric missing‐value imputation with the *missForest* package was used; this approach outperforms multiple imputation and ensures that nested models have a consistent sample size, permitting model comparison (Stekhoven, 2022). Continuous predictors were grand‐mean centred (except for time). Analyses were preregistered: https://osf.io/bn3jq.

## RESULTS

Descriptive statistics are in Table 2 and bivariate correlations are in Table S1.

**TABLE 2:** Descriptive statistics for study variables.

	Time 0	Time 1	Time 2	Time 3	Time 4	Time 5
	*M* (SD)					
COVID‐19 disruption	27.45 (6.01)	‐	‐	‐	‐	‐
Parent psychological distress (K10)	21.64 (6.40)	21.77 (5.91)	19.99 (6.05)	20.75 (6.64)	19.77 (5.87)	19.34 (6.70)
Parenting stress	4.84 (1.48)	4.62 (1.17)	3.99 (1.54)	3.72 (1.25)	2.95 (1.57)	3.54 (1.58)
Family functioning (FAD)	2.02 (0.48)	1.95 (0.43)	1.85 (0.45)	1.79 (0.49)	1.77 (0.52)	1.82 (0.51)

*Note*: For all scales: higher scores suggest greater challenges. Possible score ranges: COVID‐19 Disruption Scale scores can range from 16 to 48; K10 scores can range from 0 to 40; Parenting Stress scores can range from 1 to 7; FAD scores can range from 1 to 4. Observed score ranges: COVID‐19 Disruption = 16–42; K10 = 10–40, Parenting Stress = 1–7; FAD = 1–4. For contextual understanding of the clinical sample: At Time 0 (study baseline), 58% of the sample reported K10 scores that were within the clinical range. Similarly, at Time 0, 67% of participants reported FAD scores that were within the clinical range.

Abbreviations: FAD, Family Assessment Device; K10, Kessler Psychological Distress Scale.

### Question 1: What proportion of variability in family functioning is attributable to between‐caregiver differences versus within‐caregiver change?

The ICCs from the null model (Model 0) reflect variance in FAD scores attributable to stable between‐caregiver differences relative to total variance (see Table S2). In Model 0, 59% of the variance in family functioning was accounted for by individual caregiver differences, while 41% was due to within‐caregiver change over time (including measurement error). The statistically significant result at the individual level indicates that there were stable between‐caregiver differences in reports of family functioning, irrespective of time. Similarly, the statistically significant within‐caregiver variance demonstrates that participating caregivers experienced changes in family functioning over time (including measurement error, irrespective of between‐caregiver differences in the sample).

### Question 2: What are the trajectories of family functioning?

In Model 1 (linear fixed effect of time), the intercept reflected a level of family functioning within, but at the cusp of the non‐clinical range (*b* = 1.93, *SE* *=* 0.04, *p* < .001; see Tables 3 and 4). There was a significant and negative coefficient for time with a small effect size (*b* = −.01, *SE* *=* 0.003, *p* < .001, *ηp* ^2^ = 0.03); demonstrating that family functioning improved from baseline in the months following the intervention. All effect sizes were calculated using partial eta‐squared, which isolates the specific variance associated with each independent variable within a model, holding all others constant (Correll et al., 2021).

**TABLE 3:** Fixed effects from the multilevel model examining individual trajectories of family functioning.

Fixed effects	Model 0	Model 1	Model 2	Model 3	Model 4	Model 5	Model 6	Model 7a	Model 7b
	Null	Time‐Linear	Time‐Random slope	Time‐Quadratic	Time‐Cubic	Covariates	Predictors	Interactions‐Parenting	Interaction‐Family size
	*B* [CI]	*B* [CI]	*B* [CI]	*B* [CI]	*B* [CI]	*B* [CI]	*B* [CI]	*B* [CI]	*B* [CI]
Intercept	1.88*** [1.81, 1.95]	1.93*** [1.85, 2.00]	1.93*** [1.85, 2.00]	1.98*** [1.90, 2.05]	2.01*** [1.93, 2.08]	1.98*** [1.76, 2.20]	1.99*** [1.77, 2.21]	1.99*** [1.78, 2.21]	1.93*** [1.71, 2.15]
Time in months	‐	−0.01*** [−0.02, −0.01]	−0.01*** [−0.02, −0.01]	−0.06*** [−0.08, −0.04]	−0.13*** [−0.17, −0.08]	−0.13*** [−0.17, −0.08]	−0.13*** [−0.17, −0.08]	−0.13*** [−0.17, −0.08]	−0.11*** [−0.16, −0.06]
Time in months^2^	‐	‐	‐	0.004*** [0.003, 0.01]	0.02*** [0.01, 0.03]	0.02*** [0.01, 0.03]	0.02*** [0.01, 0.03]	0.02*** [0.01, 0.03]	0.02*** [0.01, 0.03]
Time in months^3^	‐	‐	‐	‐	−0.001** [−0.002, −0.0003]	−0.001** [−0.001, −0.0003]	−0.001** [−0.002, −0.0003]	−0.001** [−0.002, −0.0003]	−0.001** [−0.002, −0.0003]
COVID‐19 disruption	‐	‐	‐	‐	‐	0.02** [0.01, 0.03]	0.01* [0.004, 0.03]	0.01* [0.004, 0.03]	0.01* [0.003, 0.03]
More than one caregiver	‐	‐	‐	‐	‐	0.10 [−0.05, 0.25]	0.10 [−0.05, 0.25]	0.10 [−0.05, 0.25]	0.10 [−0.05, 0.25]
Number of children	‐	‐	‐	‐	‐	−0.001 [−0.10, 0.01]	−0.01 [−0.10, 0.09]	−0.01 [−0.11, 0.09]	0.02 [−0.07, 0.12]
Caregiver gender	‐	‐	‐	‐	‐	0.01 [−0.21, 0.22]	0.01 [−0.20, 0.21]	0.01 [−0.20, 0.21]	0.01 [−0.20, 0.21]
K10	‐	‐	‐	‐	‐	‐	0.01*** [0.004, 0.02]	0.01** [0.004, 0.02]	0.01*** [0.005, 0.02]
PS	‐	‐	‐	‐	‐	‐	0.03** [0.01, 0.05]	0.03* [0.000, 0.02]	0.03** [0.01, 0.06]
K10 × months	‐	‐	‐	‐	‐	‐	‐	−0.000 [−0.001, 0.001]	‐
PS × months	‐	‐	‐	‐	‐	‐	‐	0.002 [−0.002, 0.006]	‐
Number of children × months	‐	‐	‐	‐	‐	‐	‐	‐	−0.01* [−0.02, 0.002]

*Note*: Parent psychological distress, parenting stress, and baseline COVID‐19 disruption were centred at their mean.

Abbreviations: *B*, beta; CI, 95% confidence intervals; K10, Kessler Psychological Distress Scale (parent mental health); PS, parenting stress.

*p < .05, **p < .01, ***p < .001 or a statistically significant difference.

**TABLE 4:** Random effects from the multilevel model examining family trajectories and family functioning.

Random effects	Model 0	Model 1	Model 2	Model 3	Model 4	Model 5	Model 6	Model 7a	Model 7b
	Null	Time‐Linear	Time‐Random slope	Time‐Quadratic	Time‐Cubic	Covariates	Predictors	Interactions‐Parenting	Interaction‐Family size
	SD [95% CI]	SD [95% CI]	SD [95% CI]	SD [95% CI]	SD [95% CI]	SD [95% CI]	SD [95% CI]	SD [95% CI]	SD [95% CI]
Level 2 (participants)									
Random intercept	0.37* [0.32, 0.42]	0.37* [0.32, 0.42]	0.37* [0.32, 0.43]	0.37* [0.32, 0.43]	0.37* [0.32, 0.43]	0.35* [0.30, 0.40]	0.35* [0.29, 0.40]	0.35* [0.39, 0.40]	0.35* [0.29, 0.40]
Level 1 (time)									
Random intercept	0.31* [0.29, 0.33]	0.30* [0.29, 0.32]	0.28* [0.27, 0.30]	0.28* [0.26, 0.29]	0.27* [0.26, 0.29]	0.27* [0.26, 0.29]	0.27* [0.25, 0.29]	0.27* [0.25, 0.29]	0.27* [0.25, 0.29]
Random slope	‐	‐	0.02* [0.01, 0.03]	0.02* [0.02, 0.03]	0.02* [0.02, 0.03]	0.02* [0.02, 0.03]	0.02* [0.01, 0.03]	0.02* [0.01, 0.03]	0.02* [0.01, 0.03]
Correlation between Level 2 intercept and Level 1 slope	‐	‐	−0.13 [−0.41, 0.20]	−0.15 [−0.41, 0.16]	−0.15 [−0.41, 0.16]	−0.12 [−0.36, 0.24]	−0.18 [−0.41, 0.22]	−0.13 [−0.41, 0.23]	−0.11 [−0.39, 0.24]

Abbreviations: CI, 95% confidence intervals; SD, standard deviation.

*p < .05, or a statistically significant difference.

**FIGURE 1:**
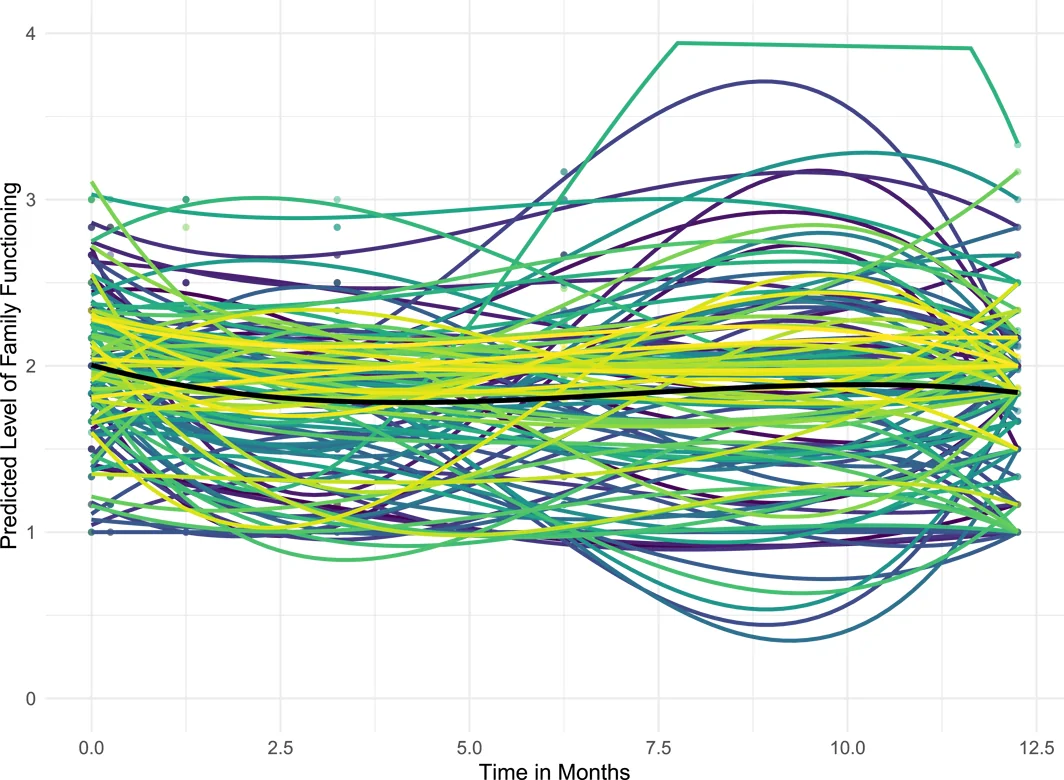
Model‐predicted individual‐level cubic trajectories of family functioning over time. The dots depict the scores of individual participants at each wave of measurement. Each coloured line represents the predicted individual trajectory for that participant. The black trend line in the centre of the graph represents the mean predicted trajectory across the sample. With the Family Assessment Device, lower scores are suggestive of higher family functioning, and higher scores are suggestive of lower family functioning.

In Model 2, time was added with a random slope (permitting separate trajectories for everyone); the random slope was significant, demonstrating that individual families differed in their trajectories of family functioning. Nelder Mead optimization was used to achieve convergence in Models 2, 3, and 5, as recommended by rrdr.io (2024); the results were substantively identical with and without this addition to model estimation. Models 3 and 4 explored the shape of the trajectory of change in family‐functioning post‐intervention. The data were consistent with a cubic (i.e., horizontal S‐shaped) trajectory (*b* = −.001, SE *=* 0.0003, *p* < .01), with a small effect size (*ηp* ^2^ = .02). This result demonstrates a trend of initial and immediate improvement post‐intervention, followed by a slight change in trajectory around 6 months post‐intervention (towards worse family functioning), which attenuated over time, and resumed on a trajectory of improvement. On average, despite the fluctuations throughout the course of the year post‐intervention, change was generally maintained over time (see Figure 1). That said, there was substantial between‐caregiver fluctuations in family functioning.

### Questions 3 and 4: What predictors are associated with family functioning?

When time‐invariant covariates were added to the model as fixed effects (Model 5), only baseline COVID‐19 disruption was significant, such that higher disruption was associated with lower baseline family functioning (*b =* .02, SE *=* 0.01*, p* = .001), with a medium effect size (*ηp* ^2^ = .09). Whether one or more caregivers participated in the workshop, the number of children in the home, and caregiver gender were not significant (*p*s > .05). In Model 6, where primary independent variables were added, both caregiver psychological distress and parenting stress for the target child were significantly associated with worse family functioning, such that higher psychological distress (*b =* .01, SE *=* 0.003*, p* < .001, *ηp* ^2^ = .02) and parenting stress (*b =* .03, SE *=* 0.01*, p* < .01, *ηp* ^2^ = .01) were both associated with lower baseline family functioning, with small effects. Finally, in Model 7a cross‐level linear interactions between Participants (Level 2) and Time (Level 1) were run to explore the relationship between these variables and their relationship to change in family functioning over time as a linear trajectory. Neither interaction was significant (*p*s > .05). Thus, the observed post‐intervention fluctuations in family functioning were not predicted by time‐varying changes in these two areas of caregiver well‐being.

#### Exploratory analyses

Other iterations of Model 7 were tested with an interaction between the linear trajectory of time and each of the covariates. In these models, the interactions in Model 7a were replaced, in order to free up model parameters. A significant interaction with a small effect size (*ηp* ^2^ = .05) was found between time and the number of children under 18 in the home. Families with more children showed a steeper slope of improvement in family functioning over time (*b =* −.01, SE *=* 0.004*, p* < .05), relative to families with fewer children (Model 7b; see Figure S1). To probe if this interaction would be maintained with a cubic trajectory of time, linear, quadratic, and cubic interactions were added to the model (removing the non‐significant covariates to free up parameters); the effect was not maintained (*p*s > .05). Whether more than one caregiver participated in the workshop, the gender of the participating caregiver, and baseline COVID‐19 distress did not significantly predict changes in family functioning over time (Models 7c–7e, *p*s > .05). Finally, target child age (Model 7f) was added to the model as a predictor and in an interaction with time, which were both non‐significant (*p*s > .05). When fit statistics were compared across models through a chi‐square likelihood ratio test, Model 7b best fit the data (Bates et al., 2015; see Tables S3 and S4).

## DISCUSSION

This study explored changes in family functioning in the year following a brief and virtual EFFT workshop for parents of children facing mental health challenges. The following insights were gleaned from the results: (1) family functioning is both a stable (between‐caregiver) and time‐varying construct, (2) families experienced positive changes in family relationships that were maintained up to 12 months following brief EFFT, and (3) parent‐specific and pandemic‐related environmental stressors are linked to family functioning irrespective of time, an family size predicted post‐intervention treatment gains.

### Family functioning is a stable and time‐varying process

Following brief virtual EFFT intervention, significant variation in family functioning was observed at the between and within caregiver levels. That is, caregivers differed from one another in their reports of interpersonal dynamics in the home, above and beyond the within‐person differences resulting from change over time and measurement error. This result suggests that family functioning is simultaneously unique to each caregiver (reporting on their family) and a flexible interpersonal process that is amenable to change. Though there are both genetic and environmental processes that inform psychological well‐being and family functioning, the study findings suggest that there are likely environmentally‐induced changes in family functioning that occurred throughout the 1‐year follow‐up period (Jami et al., 2021). Thus, in addition to being a relatively stable characteristic of families, family functioning is a time‐varying quality that is informed by caregiver‐ and child‐specific factors. Notwithstanding, perceptions of family life can differ depending on the informant, as has been demonstrated in multi‐informant studies, including those that have similarly used the Family Assessment Device (Georgiades et al., 2008; Jenkins et al., 2009; Sianko & McDonell, 2020). As the present study relied on caregiver reports, it is important for future studies of family functioning in this context to employ a multi‐informant approach, utilizing perspectives of both caregivers and children.

### EFFT as a therapeutic tool for family‐wide care

In this study, a cubic‐shaped improvement in family functioning was maintained up to 12 months following a brief, virtual EFFT intervention. This pattern reflects a trend of initial (caregiver‐reported) treatment gains in the health of family relationships, followed by a slight deterioration around 6 months post‐intervention, which attenuated and continued a trajectory of improvement. Despite these fluctuations throughout the course of the post‐intervention year, positive changes were maintained, and the reduction in treatment gains did not reach the level measured at the pre‐intervention baseline, on average. These findings overlap with the results of existing studies that have demonstrated positive family‐level outcomes following longer‐term EFFT and other emotion‐focused interventions (Havighurst et al., 2025; Otterpohl et al., 2020; Smith et al., 2023). In view of the growing evidence base for EFFT, short‐term interventions have been associated with gains in areas of well‐being that are directly targeted by the intervention (children's mental health and caregiver self‐efficacy), in addition to those that pertain to broader interpersonal dynamics, including general family functioning (Foroughe et al., 2023; Goveas et al., 2024). Additionally, the cubic trajectory of family functioning identified in this study also mirrors the pattern of post‐intervention change observed in parent self‐efficacy and children's behaviour problems observed with the same intervention, delivered in‐person (Foroughe et al., 2023). These results are consistent with the family‐wide applications of EFFT as a transdiagnostic and relationally‐focused treatment approach (LaFrance et al., 2020).

Brief EFFT was designed to support parents in learning advanced caregiving strategies to boost their sense of self‐efficacy in managing their child(ren)'s mental health concerns. This intervention could have improved caregivers' overall sense of well‐being through an enhanced sense of effectiveness and reduced parenting stress, which may have helped mitigate broad interpersonal tensions across other family relationships. Similarly, parents may have applied EFFT strategies towards their own emotions or taught them to their partner or other children in the home, effectively modelling an emotionally validating family culture (Hajal & Paley, 2020). These hypotheses align with literature that has demonstrated an association between lower family functioning and health‐related quality of life in parents of children with a mental health challenge (Reed et al., 2023). Thus, the broader interpersonal climate of the family system may have indirectly benefitted from this parenting intervention, in much of the same way that the intervention was expected to benefit focal child outcomes (mental health symptoms), despite that children did not directly participate in treatment (Zahl‐Olsen et al., 2023). Overall, these findings are consistent with the goals of EFFT, which is intended to galvanize the parent‐child relationship as a vehicle for both relational recovery and the amelioration of child mental health concerns (LaFrance et al., 2020).

### Family‐wide factors in treatment

In this study, families who were experiencing a higher level of disruption due to COVID‐19 demonstrated lower baseline family functioning. This result overlaps with findings from a meta‐analysis on parent well‐being and a systematic review of the COVID‐19 family disruption model (Racine et al., 2022; Shoychet et al., 2023). Collectively, these reviews have suggested that pandemic‐induced disruptions in parental mental health may reflect broad changes in relational dynamics at the family‐level, which in turn, created an environment where families lacked the necessary support and resources to maintain well‐being in the face of chronic and multisystemic strain. Interestingly, whether families had more than one caregiver participating in the workshop and the gender of the caregiver did not predict baseline or post‐intervention family functioning. It may be that in dual‐caregiver families with only one parent participating, that caregiver may have modelled or shared the principles and resources they acquired with their partner, effectively multiplying the impact of the intervention.

The observed interaction between family size and family functioning over time may reflect positive contagion as caregivers began to implement advanced skills (Canzi et al., 2021). Aligned with family systems theory and family functioning research during the pandemic, if caregivers of larger families applied EFFT techniques in the home, their modelling would have been observed not only by the target child but also their siblings, increasing the potential ‘ports of entry’ by which this intervention could have strengthened family relationships (Canzi et al., 2021; Nelson et al., 2009; Sameroff, 2005). Finally, the absence of significant gender differences could suggest that all caregivers experienced similar gains in family functioning following the intervention, or it could also reflect the underrepresentation of male and gender minority caregivers (12%). Previous research has demonstrated smaller effect sizes in outcomes for fathers compared to mothers following parent interventions, a challenge which may be partially due to the general overrepresentation of mothers in programs of this nature and parenting research more generally (Everett et al., 2021; Tully et al., 2017).

Finally, parenting stress and caregiver distress did not significantly predict patterns of change in family functioning following the intervention, demonstrating that family relationships improved across the sample regardless of parents' ongoing level of reported challenge in these areas. It is possible that the nature of the intervention, which is sensitive to the role of caregiver mental health and stress in the promotion of family‐wide change, was indeed equally effective in supporting all families. Relatedly, the study period was during a historical time where there was an unusually high amount of stress for families, thereby attenuating the capacity for these factors to modify trajectories. Conversely, it could be that there were such sizable main effects on baseline family functioning, leaving little room for these variables to serve as moderators. At the same time, there was substantial variability in caregiver‐reported trajectories of family functioning that was not explained by the variables included in this study. Future researchers might consider exploring other processes that may account for differential responses following brief EFFT, including dynamic modelling techniques that can identify stochastic change and sudden gains. Notwithstanding, these results still affirm the family stress and systems theory frameworks, demonstrating that the interpersonal climate in the family is co‐constructed by interpersonal dynamics (parent‐child interactions and parenting stress), individual factors (caregiver psychological distress), and the broader family‐wide environment (COVID‐19 disruption and family size) in the year following EFFT interventions (Cox & Paley, 2003; Masarik & Conger, 2017).

### Future directions

A primary limitation of this study is the absence of a control group. Though this design precludes conclusions about the effectiveness of brief EFFT compared to receiving no other treatment, given the timing of the project during the COVID‐19 pandemic, maximization of mental health services was prioritized and the first randomized controlled trial of EFFT is now underway by other researchers (Seddon et al., 2025). Furthermore, this study employed a sophisticated analytical approach, which allowed for disentangling between versus within caregiver variance in family functioning. Future studies might use an observational design and/or multi‐informant perspective, which would mitigate challenges associated with caregiver self‐report of family‐wide outcomes and single‐informant bias, while also allowing for the disentangling of variance at both the individual and family level. Finally, the researchers acknowledge other threats to internal validity, including period effects (e.g., improvements related to lessening pandemic restrictions) and developmental maturation (i.e., children getting older), which were partially accounted for by including baseline COVID‐19 disruption and an age‐by‐time interaction in the analyses.

Amidst calls for research in the area of family functioning within children's mental health care, evaluations of family‐wide outcomes have been more limited, due to the challenges inherent to longitudinal, multi‐level family assessment (Buka et al., 2022; Everett et al., 2021; Georgiades et al., 2008; Lewandowski et al., 2010; McCarthy & Guerin, 2021; Tang et al., 2024). This study contributes to the growing evidence base across these domains, demonstrating that interventions designed to target child mental health through the parent‐child relationship are associated with psychosocial benefits for the larger family system (which is, itself, correlated with parent well‐being). Thus, a primary application of these findings in clinical practice includes consideration of family‐wide factors within case formulation and treatment planning for children's mental health concerns. Practically, this also includes consideration of whether short‐term emotion‐focused parenting interventions, such as brief EFFT, may be valuable as an adjunctive treatment.

## CONCLUSION

These results extend existing literature in the areas of family functioning, EFFT, and brief parenting interventions, demonstrating that targeting the co‐parent and parent‐child relationships, as well as caregiving skills offers distal interpersonal benefits for the broader family system (family functioning). Comparable results have been observed through virtual and in‐person service delivery for brief EFFT, demonstrating that this program can be flexibly applied across contexts. This work demonstrates the value of a family‐wide lens when evaluating parenting interventions, and strengthens the growing evidence that brief EFFT is a valuable, cost‐ and resource‐efficient approach, aligned with identified service needs in children's mental health care (Goveas et al., 2024).

## AUTHOR CONTRIBUTIONS

**Laura Colucci**: Conceptualization; funding acquisition; writing—original draft; writing—review and editing; formal analysis; visualization; project administration; data curation. **Mirisse Foroughe**: Methodology; data curation; supervision; conceptualization; writing—review and editing; project administration; resources. **Imogen M. Sloss**: Data curation; writing—review and editing; resources. **Dillon T. Browne**: Writing—review and editing; methodology; conceptualization; investigation; funding acquisition; project administration; supervision; data curation.

## CONFLICT OF INTEREST STATEMENT

The authors declare no conflicts of interest.

## ETHICAL CONSIDERATIONS

Informed consent was appropriately obtained from all participants. This study received ethics clearance through the University of Waterloo Ethics Committee on 25 August 2020; approval reference number: #41875.

## Supporting information

Supporting Information S1

## Data Availability

Research data are not shared as not all participants consented to the use of their study data for future research unrelated to this project.
