# Supplementary material for: Family functioning following a brief, virtual emotion‐focused family therapy intervention for children's mental health
Source: JCPP Adv. 2026 Jan 13;6(3):e70074. doi: 10.1002/jcv2.70074 (PMC13339665; doi:10.1002/jcv2.70074)
Supplement: Supplementary file 1 [file JCV2-6-e70074-s001.docx]

**Family Functioning following a Brief, Virtual Emotion-Focused Family Therapy Intervention for Children’s Mental Health**

**Supporting Information**

**Appendix S1. Atypical Participation Analyses**

Amongst participants in the study, a few participants were flagged for atypical participation in the intervention (n=15). These included those that did not complete the entire workshop or watched a partial recording at a second date, a second caregiver in the family participating in the workshop several months later (resulting in a new “dose” of treatment mid-way through the initial caregiver’s survey data), and individuals who were no longer in communication with their child and/or dropped out of the study after baseline. When a one-way ANOVA was run with the full sample (n=159) to explore if there were mean differences between the “typical” group and those with imperfect participation across relevant study variables (FAD, K10, and parenting stress for the target child), non-significant differences were observed across all variables, with the exception of the parenting stress variable at Time 2 (*F*(1, 143) = 8.37, *p = .*05) and Time 3 (*F*(1, 140) = 7.31, *p = .*04), where the atypical group reported slightly lower levels of parenting stress across both time points (Time 2: *M* =2.86, *SD* = 1.77 vs *M* = 3.98, *SD* = 1.47; Time 3: *M* =2.67, *SD* = 1.21 vs *M* = 3.79, *SD* = 1.29). Additionally, there were a handful of participants whose responses were flagged due to failing one or more “attention checks” in the surveys (n = 15). When a one-way ANOVA was run to explore if there were mean differences between the “typical” group and those with failed attention checks, non-significant differences were observed across all variables (*p*’s >.05). All of the above-described cases were retained within the dataset.

**Appendix S2. Sensitivity Analyses for Age and Data Non-Independence**

The analysis was completed in two stages: First, given the large age range for target children (1-29 years) represented in this sample, an age-filter was applied to the data, and the analyses were run including only participants with children ages 5-25 years of age, and with only one caregiver representing each family (n = 117). Consistent with the approach taken by Sloss and colleagues (2025), this restricted age-range was selected in view of research that suggests that the age of adolescence should include youth up to 25 years of age and consideration that the role of EFFT techniques in the family system operate differently for infants and toddlers (Sawyer et al., 2018). Subsequently, analyses were then run with the full sample, including children of any age and additional caregivers nested within the same family (n=158). This was done to avoid estimating a cross-classified multilevel model, which would significantly increase complexity, but only apply to 35 of the respondents.

Across both samples, the pattern of results was the same and when the intraclass correlations (ICCs) were compared between samples, the confidence intervals overlapped, suggesting that adding these participants did not result in statistically significant differences in the degree of variance accounted for by each level of the model. Finally, when model fit statistics were compared, the pattern was consistent across samples. This is likely attributable to most children being around 12.44 years old (*SD* = 4.80), and very few youths on extreme tails of the age distribution. In order to prioritize precision of estimates (which could be biased by adding dual caregivers from the same family) and generalizability of the study findings to the age group of primary interest, the results representing families of target children aged 5-25 years (n=117) have been presented in all tables and discussions of the results.

**Appendix S3. Further Discussion of Analytical Strategy**

Multilevel modelling can be likened to a complex regression model and offers the capacity to statistically model data with a nested structure (National Centre for Research Methods, 2021). The value of multi-level modelling in family process research is that it permits a disentangling of within- and between-family differences over time (Lin et al., 2021). For a thorough discussion of between- vs within-family processes and associated statistical methods, see Browne and colleagues (2019).

Multi-level modelling has been applied in the context of family research by O’Connor and colleagues (2006) and Jenkins and colleagues (2009), when evaluating differential parenting and emotional problems in children (respectively). Using both cross-classified and standard multi-level models, Jenkins and colleagues (2009) were able to identify differences between family-specific and child-specific family processes; relatedly, this approach also enabled an exploration of differences between siblings in the same family. Similarly, O’Connor and colleagues (2006) demonstrated that this type of analysis can be used to ascertain the level of variance in outcomes that is accounted for by different family subsystems. In this study, the models were not cross-classified due to the limited number of families where two caregivers participated in the study. Additional justification for this approach is also discussed by Meunier and colleagues (2005).

Finally, growth curve modelling enables an exploration of the overall trajectory or “pathway” of change across longitudinal timepoints. This analytic approach tests linear and subsequent higher-order functions in a step-by-step fashion (i.e., a linear model, followed by a quadradic model, followed by a cubic model). With each subsequent model, the highest-order function that is found to be significant is considered the “trajectory” of the specified outcome (which in this case, is family functioning). For a detailed overview of this approach, see Curran and colleagues (2010).

**Appendix S4. Additional Details about the EFFT Intervention**

Several didactic and hands-on components were used to support learning during the workshops including psychoeducation, audiovisual aids, takeaway handouts for parents, one-on-one coaching (in breakout rooms and for observation by other attending parents), role-plays, peer-support, and opportunities for questions. Further, for a more detailed explanation of each of the EFFT components, see LaFrance and colleagues (2020) and Seddon and colleagues (2025).

In terms of existing literature for virtual EFFT, a recently published case study by Smith and colleagues (2023) demonstrated positive outcomes from an eight-week virtual EFFT intervention. Between pre-post intervention, the participating parents demonstrated improvements across multiple levels of family well-being, including general family functioning, parental self-efficacy, parent psychopathology, and child depression, anger, and anxiety symptoms (Smith et al., 2023).

At the time of writing, this study (second to Sloss et al., 2025) represented some of the first research demonstrating support for virtually-delivered, brief EFFT interventions, and among a limited few in this domain that have evaluated family-level outcomes. The presented findings highlight the feasibility of service delivery within this modality, demonstrate the value of whole-family research approaches following these types of interventions, and support the need for additional research in this area (e.g., with a larger sample and across EFFT intervention formats, such as brief 2-day workshops and longer-term EFFT).

**References**

Browne, D. T., Leckie, G., & Jenkins, J. M. (2019). Chapter 19: Understanding Couple and	Family Dynamics Through Dyadic Methodology. In B. H. Fiese (Ed.), *APA Handbook of	Contemporary Family Psychology: Vol. 1. Foundations, Methods, and Contemporary	Issues Across the Lifespan.* American Psychological Association.

Curran, P. J., Obeidat, K., & Losardo, D. (2010). Twelve frequently asked questions about	growth curve modeling. *Journal of Cognition and Development, 11*(2), 121-136.	https://doi.org/10.1080/15248371003699969

Lafrance, A., Henderson, K. A., & Mayman, S. (2020). *Emotion-focused family therapy: A	transdiagnostic model for caregiver-focused interventions.* American Psychological	Association.

Lin, S., Schleider, J. L., & Eaton, N. R. (2020). Family processes and child psychopathology: A	between- and within-family/child analysis. *Research on Child and Adolescent	Psychopathology, 49*, 283-295. https://doi.org/10.31219/osf.io/cwyah

Jenkins, J. M., Cheung, C., Frampton, K., Rasbash, J., Boyle, M. H., & Georgiades, K. (2009).	The use of multilevel modeling for the investigation of family process. *International	Journal of Developmental Science, 3*(2), 131-149. https://doi.org/10.3233/dev-2009-3204

National Centre for Research Methods (NCRM). (2021, June 17). *Cross Classified Models	Part 1: Introduction* [Video]. YouTube.	https://www.youtube.com/watch?v=jOZZYksVty4

O'Connor, T. G., Dunn, J., Jenkins, J. M., & Rasbash, J. (2006). Predictors of between-family	and within-family variation in parent-child relationships. *Journal of Child Psychology	and Psychiatry, 47*(5), 498-510. https://doi.org/10.1111/j.1469-7610.2005.01527.x

Sawyer, S. M., Azzopardi, P. S., Wickremarathne, D., & Patton, G. C. (2018). The age of	adolescence. The Lancet. *Child & Adolescent Health, 2*(3), 223–228.	<https://doi.org/10.1016/S2352-4642(18)30022-1>

Seddon, J.A., Reaume, C.L. & Thomassin, K. A six-week group program of emotion focused	family therapy for parents of children with mental health challenges: Protocol for a	randomized controlled trial. *BMC Psychiatry 25*, 131 (2025).	https://doi.org/10.1186/s12888-024-06382-y

Sloss, I., Smith, J., Colucci, L., Foroughe, M., Browne, D., (2025). Trajectories of Child &	Caregiver Positive Coping Following a Brief Emotion-Focused Family Therapy (EFFT)	Intervention. *Journal of Marital and Family Therapy, 51*(3)*,* e70029.	https://doi.org/10.1111/jmft.70029.

**Tables**

**Table S1**

*Bivariate Correlations between Study Variables*

|  | >1CG | Kids | Gender | FAD-T0 | FAD-T1 | FAD-T2 | FAD-T3 | FAD-T4 | FAD-T5 | K10-T0 | K10-T1 | K10-T2 | K10-T3 | K10-T4 | K10-T5 | PS-T0 | PS-T1 | PS-T2 | PS-T3 | PS-T4 | PS-T5 |
| --- | --- | --- | --- | --- | --- | --- | --- | --- | --- | --- | --- | --- | --- | --- | --- | --- | --- | --- | --- | --- | --- |
| COVID | -.05 | .06 | .03 | .34^**^ | .23^*^ | .21^*^ | .24^*^ | .10 | .11 | .38^**^ | .35^**^ | .37^**^ | .42^**^ | .30^**^ | .33^**^ | .10 | .11 | -.02 | .11 | -.07 | -.00 |
| >1CG | - | -.13 | .19^*^ | .15 | .02 | .10 | .15 | .08 | .09 | -.04 | .05 | .06 | -.12 | -.03 | -.15 | .12 | -.05 | .01 | .00 | .18 | .16 |
| Kids | - | - | -.22^*^ | -.01 | -.03 | .02 | .02 | -.04 | -.14 | .09 | .12 | .08 | .12 | .09 | .13 | -.04 | .00 | -.19 | -.02 | -.08 | -.03 |
| Gender | - | - | - | -.01 | .10 | .02 | -.03 | -.11 | .03 | -.02 | -.01 | .04 | -.05 | -.07 | -.03 | .01 | .01 | -.06 | .08 | -.01 | -.02 |
| FAD-T0 | - | - | - | *-* | .66^**^ | .63^**^ | .58^**^ | .53^**^ | .45^**^ | .35^**^ | .34^**^ | .34^**^ | .20^*^ | .22^*^ | .27^**^ | -.01 | .18 | -.00 | .12 | .13 | .09 |
| FAD-T1 | - | - | - | - | - | .65^**^ | .66^**^ | .63^**^ | .51^**^ | .22^*^ | .24^*^ | .16 | .10 | .14 | .23^*^ | -.10 | .18 | -.09 | .12 | .16 | .06 |
| FAD-T2 | - | - | - | - | - | *-* | .69^**^ | .59^**^ | .56^**^ | .14 | .21^*^ | .31^**^ | .14 | .18 | .23^*^ | -.08 | .13 | .13 | .18 | .17 | .09 |
| FAD-T3 | - | - | - | - | - | - | - | .52^**^ | .52^**^ | .29^**^ | .29^**^ | .31^**^ | .16 | .18 | .21^*^ | -.08 | .14 | -.11 | .06 | .15 | .07 |
| FAD-T4 | - | - | - | - | - | - | - | *-* | .73^**^ | .21^*^ | .16 | .27^**^ | .21^*^ | .31^**^ | .24^*^ | -.04 | .07 | .05 | .22^*^ | .27^**^ | .25^*^ |
| FAD-T4 | - | - | - | - | - | - | - | - | - | .12 | .11 | .21^*^ | .14 | .14 | .25^*^ | -.03 | .07 | .02 | .08 | .16 | .23^*^ |
| K10-T0 | - | - | - | - | - | - | - | - | - | *-* | .84^**^ | .65^**^ | .57^**^ | .57^**^ | .57^**^ | .18 | .23^*^ | .08 | .24^*^ | .15 | .20^*^ |
| K10-T1 | - | - | - | - | - | - | - | - | - | - | - | .70^**^ | .54^**^ | .64^**^ | .60^**^ | .21^*^ | .25^**^ | .08 | .21^*^ | .13 | .20^*^ |
| K10-T2 | - | - | - | - | - | - | - | - | - | - | - | *-* | .62^**^ | .58^**^ | .59^**^ | .17 | .15 | .24^*^ | .20^*^ | .10 | .13 |
| K10-T3 | - | - | - | - | - | - | - | - | - | - | - | - | - | .67^**^ | .66^**^ | .12 | .15 | .23^*^ | .42^**^ | .10 | .24^*^ |
| K10-T4 | - | - | - | - | - | - | - | - | - | - | - | - | - | *-* | .69^**^ | .17 | .10 | .17 | .38^**^ | .27^**^ | .30^**^ |
| K10-T5 | - | - | - | - | - | - | - | - | - | - | - | - | - | - | - | .14 | .12 | .09 | .31^**^ | .17 | .28^**^ |
| PS-T0 | - | - | - | - | - | - | - | - | - | - | - | - | - | - | - | *-* | .47^**^ | .40^**^ | .35^**^ | .37^**^ | .31^**^ |
| PS-T1 | - | - | - | - | - | - | - | - | - | - | - | - | - | - | - | - | - | .40^**^ | .39^**^ | .35^**^ | .19 |
| PS-T2 | - | - | - | - | - | - | - | - | - | - | - | - | - | - | - | - | - | *-* | .56^**^ | .36^**^ | .24^*^ |
| PS-T3 | - | - | - | - | - | - | - | - | - | - | - | - | - | - | - | - | - | - | - | .50^**^ | .55^**^ |
| PS-T4 | - | - | - | - | - | - | - | - | - | - | - | - | - | - | - | - | - | - | - | - | .66^**^ |

*Note.* >1CG = More than one caregiver participating in the workshop, Kids = Number of children in the home, Gender = Caregiver gender, COVID = Baseline COVID-19 Disruption, FAD = Family Assessment Device, K10 = Caregiver Distress, PS = Parenting Stress for the Target Child.
**p* < .05 ***p* < .01 or a statistically significant difference.

**Table S2**

*Intraclass Correlation Coefficients (ICCs) of the Multilevel Model Output Examining Individual Trajectories of Family Functioning*

| *Variance (ICC)* | **Model 0**  Null | **Model 1**  Time - Linear | **Model 2**  Time - Random Slope | **Model 3**  Time - Quadratic | **Model 4**  Time - Cubic | **Model 5**  Covariates | **Model 6**  Predictors | **Model 7a**  Interactions - Parenting | **Model 7b**  Interaction - Family Size |
| --- | --- | --- | --- | --- | --- | --- | --- | --- | --- |
| Level 2 - Individual | 59%* | 60%* | 64%* | 66%* | 66%* | 65%* | 64%* | 64%* | 64%* |
| Level 1 - Time | 41%* | 40%* | 36%* | 34%* | 34%* | 35%* | 36%* | 36%* | 36%* |

*Note:* Adjusted ICCs reported.

**Table S3**

*Model Fit Statistics from Multilevel Continuous Growth Model Output Examining Individual Trajectories and Family Functioning*

| *Model No./ Fit Statistic* | **Model 0**  Null | **Model 1**  Time - Linear | **Model 2** Time -  Random Slope | **Model 3**  Time - Quadratic | **Model 4**  Time - Cubic | **Model 5** Covariates | **Model 6** Predictor | **Model 7a**°  Interactions - Parenting | **Model 7b**° Interaction - Family Size |
| --- | --- | --- | --- | --- | --- | --- | --- | --- | --- |
| AIC | 603.21 | 586.79 | 576.96 | 549.68 | 542.90 | 538.39 | 515.26 | 518.36 | 511.82 |
| BIC | 616.87 | 605.01 | 604.29 | 581.55 | 579.33 | 593.03 | 579.01 | 591.22 | 580.13 |
| Log-likelihood | -298.60 | -289.39 | -282.48 | -267.84 | -263.45 | -257.19 | -243.63 | -243.18 | -240.91 |
| ***χ²*** | - | 18.42 | 13.82 | 29.29 | 8.77 | 12.51 | 27.13 | 0.89 | 5.43 |
| *df* | - | 1 | 2 | 1 | 1 | 4 | 2 | 1 | 1 |
| *p* | - | <.001*** | <.001*** | <.001*** | 0.003** | 0.013* | <.001*** | 0.64 | 0.02* |
| Number of Parameters | 3 | 4 | 6 | 7 | 8 | 12 | 14 | 16 | 15 |

*Note.* AIC - Akaike's Information Criterion, BIC = Bayesian Information Criterion. For interpretation: Lower AIC and BIC values indicate a better fit, as do higher Log Likelihood values. *df* represents the number of additional parameters added to the current model, compared to the previous nested model.
°Statistics for both versions of Model 7 respectively reflect comparisons between Model 6 and 7a, and Model 6 and 7b, given that Model 7a and Model 7b are not fully nested. * *p* < .05, ***p* < .01, *** *p* < .001, or a “statistically significant difference.”

**Table S4**

*Model Fit Statistics from Multilevel Continuous Growth Model Output Examining Individual Trajectories and Family Functioning*

| *Model No./ Fit Statistic* | **Model 7c**°  Interaction - More than One Caregiver | **Model 7d** °  Interaction -  Caregiver Gender | **Model 7e**°  Interaction -  COVID-19 | **Model 7f** °  Interaction -  Child Age |
| --- | --- | --- | --- | --- |
| AIC | 517.25 | 517.20 | 514.78 | 513.13 |
| BIC | 585.56 | 585.51 | 583.09 | 585.99 |
| Log-likelihood | -243.62 | -243.60 | -242.39 | -240.56 |
| ***χ²*** | 0.01 | 0.06 | 2.47 | 6.13 |
| *df* | 1 | 1 | 1 | 2 |
| *p* | 0.93 | 0.81 | 0.12 | 0.047* |
| Number of Parameters | 15 | 15 | 15 | 15 |

*Note.* In each of the above models, each specified variable was added to the model as an interaction with linear time. AIC - Akaike's Information Criterion, BIC = Bayesian Information Criterion. For interpretation: Lower AIC and BIC values indicate a better fit, as do higher Log Likelihood values. *df* represents the number of additional parameters added to the current model, compared to the previous nested model. Chi-square statistics for all versions of Model 7 respectively reflect comparisons between Model 6 and the selected model, given that these iterations of Model 7 (Models 7c-7f) are not fully nested. * *p* < .05, ***p* < .01, *** *p* < .001, or a “statistically significant difference.”

**Figures**

**Figure S1**

*Interaction between Time and the Number of Children in the Family on Trajectories of Family Functioning over Time*

Number of children in the home: 1 child, 2 children, 3 children, 4 children

*Note.* For the Family Assessment Device (FAD), scores range from 0-4; lower scores are suggestive of higher family functioning, and higher scores are suggestive of lower family functioning.
